# Federated Flight Anomaly Detection with Self-Supervised Forecasting: A Flight-Disjoint Evaluation

**DOI:** 10.3390/s26206376

**Published:** 2026-10-09

**Authors:** Anıl Sezgin

**Affiliations:** 1 Department of Computer Engineering, Istanbul Arel University, Istanbul 34537, Türkiye; anilsezgin@arel.edu.tr; 2 Research and Development, Hypersense, Istanbul 34212, Türkiye

**Keywords:** UAV, UAV anomaly detection, federated learning, self-supervised learning, flight telemetry, fault detection

## Abstract

Reliable anomaly detection is essential for the safe operation of unmanned aerial vehicles (UAVs), particularly in distributed settings where flight telemetry is generated across multiple vehicles or operational sessions and cannot always be centrally collected. This work investigates UAV anomaly detection using models capable of generalizing to unseen flights while keeping raw telemetry within the simulated client partitions during federated training. A self-supervised forecasting framework is evaluated under a simulated federated partitioning of the ALFA corpus and benchmarked using local training, Federated Averaging (FedAvg), FedProx, personalized FedAvg, and centralized learning under a strictly flight-disjoint evaluation protocol. The evaluation comprises 47 complete flights, including 36 failed and 11 nominal flights, grouped into 13 heterogeneous session-based federated clients that serve as statistical partitions rather than physically independent UAVs. The resulting federated setting exhibits non-IID characteristics due to differences in local flight composition and data volume across clients. Federated training is performed for 20 communication rounds with two local epochs per round, and evaluation uses three-fold flight-disjoint cross-validation. Complete flights, including failed flights, are assigned exclusively to either the training or held-out test partition within each fold, ensuring that no samples or overlapping windows from a test flight contribute to model training. An LSTM learns nominal flight dynamics through multivariate next-step forecasting, and the resulting forecasting error is converted into an anomaly score. FedAvg achieves an area under the receiver operating characteristic curve (AUC) of 0.908 and detects 83.3% of failed flights, performing competitively with centralized learning, which obtains an AUC of 0.904 and an 84.4% detection rate. Beyond discrimination performance, calibration, threshold scope, temporal persistence, detection delay, and false-alarm burden are also evaluated. With rolling calibration and a global threshold, the detector maintains an 87.2% flight-level detection rate with a median detection delay of 0.72 s while reducing the false-alarm burden from 202.1 to 66.0 events per hour. However, this alarm frequency remains too high for unattended operational deployment. A more conservative configuration reduces the burden to 8.17 events per hour while retaining 82.8% failed-flight detection and achieving an event precision of 0.851, although the median detection delay increases to 3.38 s. Within this session-partitioned experimental setting, parameter aggregation recovers most of the performance loss associated with local-only training and approaches centralized performance without pooling raw telemetry. These findings should be interpreted as evidence for distributed model aggregation under statistical data partitioning rather than as validation of a fully deployed federated UAV network, while the operational analysis further shows that strong discrimination and flight-level detection do not necessarily translate into practically acceptable alarm behavior.

## 1. Introduction

UAVs are increasingly utilized in applications such as inspection, mapping, logistics, and safety-critical monitoring, where reliable operation requires the timely identification of abnormal system behavior [1]. In these applications, faults or deviations in flight dynamics can affect sensing, actuation, and control processes. Consequently, data-driven anomaly detection is attractive because it can exploit multivariate telemetry without requiring an accurate analytical model of each airframe, payload, and operating scenario [2]. Centralized machine learning, in turn, requires raw flight logs to be transferred to a common training repository. Such a requirement may be undesirable when telemetry contains mission-specific information, communication resources are constrained, or vehicles belong to different organizations [3].

Federated learning provides an alternative distributed-learning paradigm in which participating clients train models on locally available data while model updates, rather than raw observations, are exchanged for aggregation [4]. This paradigm is particularly relevant to distributed UAV monitoring, where telemetry may originate from heterogeneous vehicles, missions, or operating conditions. In the present study, the privacy-related benefit of federated learning specifically refers to keeping raw telemetry local; parameter sharing itself is not assumed to provide formal protection against inference attacks [5]. In parallel, recent UAV health-monitoring research has increasingly considered data-driven anomaly and fault detection based on multivariate flight telemetry. Among these approaches, recurrent architectures such as LSTM networks are particularly suitable for modeling temporal dependencies in flight data, where anomalous behavior may emerge as evolving changes across multiple telemetry channels rather than isolated sensor outliers [6].

Despite these advances, an important methodological gap remains between predictive anomaly detection performance and evaluation under realistic flight-level generalization conditions. Existing telemetry-based anomaly detection studies commonly emphasize discrimination-oriented performance, whereas less attention has been given to whether learned anomaly representations generalize to entirely unseen flights [2,6]. This distinction is particularly important for UAV telemetry because sliding-window segmentation produces strongly correlated and potentially overlapping samples from the same underlying flight trajectory. If windows originating from the same flight are distributed across training and test partitions, the resulting evaluation may partially reflect flight-specific information reuse rather than generalization to independent missions [7]. Moreover, favorable anomaly score discrimination does not necessarily imply useful warning behavior in practice. Therefore, federated UAV anomaly detection should be evaluated not only in terms of predictive discrimination but also with respect to flight-level generalization, detection latency, and false-alarm behavior.

These limitations motivate two questions addressed in this study. First, can parameter aggregation across session-based statistical partitions retain performance comparable to centralized learning when complete flights, rather than randomly selected telemetry windows, are held out for evaluation? Second, does favorable anomaly discrimination translate into operationally useful behavior when detection probability, detection delay, and false-alarm frequency are considered jointly? Addressing these questions requires an evaluation protocol that prevents flight-specific information from leaking across training and test partitions and that separates ranking performance from alarm-system performance.

A suitable evaluation of this problem requires flight telemetry in which failures occur within complete temporal flight trajectories and their onset can be evaluated without fragmenting individual missions across training and test partitions. The ALFA corpus provides such a setting [8] by combining multivariate UAV telemetry with multiple flight-control failure scenarios, enabling anomaly discrimination, detection latency, and false-alarm behavior to be examined at the flight level.

This study investigates self-supervised flight anomaly detection under a simulated federated setting constructed from session-based statistical partitions of the AirLab Failure and Anomaly flight corpus. The detector learns to forecast the next telemetry sample using only failure-free local data, and the prediction error is converted into an anomaly score. Local training, Federated Averaging, FedProx, personalized FedAvg, and centralized training are compared under a strictly flight-disjoint protocol.

The federated component of this study should be interpreted as a controlled statistical-decentralization experiment rather than a full systems-level federated UAV deployment. The session-based clients emulate partitioned local telemetry stores, while communication latency, bandwidth limitations, asynchronous aggregation, heterogeneous onboard hardware, and physically independent UAV operators are outside the scope of the present evaluation.

The contribution of this study is threefold. First, we establish a leakage-resistant, flight-disjoint evaluation protocol for UAV anomaly detection under session-partitioned distributed training, ensuring that every window derived from a held-out flight remains exclusively in the test partition and thereby measuring generalization to genuinely unseen flights. Second, we move beyond window-level discrimination metrics by jointly evaluating flight-level detection probability, time-to-detection, and false-alarm events per hour, separating statistical ranking quality from operational alarm behavior. Third, we systematically examine score calibration, global versus client-specific thresholds, potentially leaky autopilot-derived channels, and temporal alarm persistence to characterize the detection–delay–false-alarm trade-off. The resulting analysis supports a deliberately limited conclusion: under the session-partitioned setting considered here, federated parameter aggregation can approach centralized performance without pooling raw flight telemetry, but this result should not be interpreted as validation of a complete federated UAV deployment, and high AUC alone is insufficient evidence of operational readiness.

The rest of this paper is structured as follows. Section 2 reviews the existing literature on UAV anomaly detection, federated learning, and telemetry-based fault detection. Section 3 explains our proposed approach, which covers a flight-disjoint evaluation method, a federated learning algorithm, calibration of the anomaly score, and alarm evaluation methodology. Section 4 reports the obtained results, which include the comparison of federated and centralized learning approaches as well as the study of trade-offs between detection, delay, and false alarms. Section 5 contains a discussion of the obtained results and the potential drawbacks of the proposed approach. The conclusion is provided in Section 6.

## 2. Related Work

Recent research relevant to the present study can be organized into four closely connected directions: telemetry-driven UAV anomaly and fault detection, federated learning for UAV and aviation systems, privacy and robustness in federated UAV networks, and deployment-oriented reliability of distributed aerial systems. Although these areas have advanced rapidly, they have largely evolved around model-level predictive performance, communication efficiency, security, or mission optimization. Comparatively less attention has been given to how federated anomaly detectors generalize to entirely unseen flights and how discrimination performance translates into operational alarm behavior. The following subsections position the present work with respect to these research directions.

### 2.1. Telemetry-Driven UAV Anomaly and Fault Detection

Recent UAV health-monitoring research increasingly treats flight telemetry as multivariate temporal data rather than as a collection of independent sensor measurements. Study [2] combined B-spline telemetry reconstruction with an LSTM autoencoder and Isolation Forest to support real-time fault detection on resource-constrained UAV edge platforms, demonstrating the importance of both temporal modeling and deployability in onboard monitoring. Study [9] modeled interactions among flight parameters through a causality-enhanced graph neural network and demonstrated anomaly detection across multiple UAV fault categories, illustrating the value of explicitly representing dependencies among telemetry channels. Study [6] compared LSTM, GRU, CNN, Transformer, and attention-enhanced recurrent architectures on real flight data and showed that temporal deep-learning architectures can identify anomalies that are difficult to capture through conventional flight-data monitoring rules.

Beyond purely temporal representations, recent work has increasingly exploited multiple feature domains. Study [10] fused time- and frequency-domain information to construct fine-grained UAV anomaly representations, addressing limitations of reconstruction-error-only detection when different abnormal patterns produce similar residual magnitudes. Study [11] instead investigated acoustic signatures of UAV faults using wavelet scattering and deep learning, showing that fault-related information can also be extracted from non-telemetry sensing modalities. Study [12] proposed the STE-RS framework to combine spatiotemporal feature learning with residual smoothing for robust UAV flight-data anomaly detection, further emphasizing the importance of modeling both temporal structure and noise characteristics.

Prediction-based approaches have special significance in relation to anomaly detection since they provide a description of nominal dynamics and deviation from expected dynamics in the future. In [13], the detection of GPS spoofing is considered in terms of trajectory prediction, where the deviation between the predicted trajectory and observed trajectory helps to reveal navigation anomalies. Study [14] studied the vibration-based health monitoring of UAVs using MEMS sensors, where the goal was to distinguish the propeller fault from the structural fault. The difference in nominal dynamic behavior can be used as an indicator of the faults in such cases. Study [15] combined the 1D Convolutional Feature Extraction technique with the Informer model to diagnose UAV bearing faults, showing the growing usage of sequence models for equipment-level health monitoring of the aerial system. Study [16] focused on electric propulsion aircraft propeller fault detection using motor signals and semi-supervised learning based on a generative model.

From the fault diagnostics literature in general, one can observe a transition from the conventional model-based approach to the data-driven and hybrid approaches. Study [17] reviews current methods of fault diagnosis and detection for unmanned aerial vehicles (UAVs), highlighting the increasing significance of machine learning and deep learning approaches used in diagnosing the nonlinear nature of UAVs. In [18], a modified LSTM GAN is suggested to solve the problem of time series anomaly detection, demonstrating how recurrent forecasting and generative learning can be combined in order to detect temporal anomalies. In [19], the uncertainty-aware hierarchical consistency modeling for UAV fault diagnosis is introduced, implying that reliable detection depends more on prediction confidence than the deterministic class output.

An operational reliability study constitutes another context within which the telemetry anomalies may be detected. In [3], the autonomous UAV agents were analyzed via scenario-based missions’ reliability analysis, where it was noted that the results should be ultimately judged based on their mission reliability. In [20], genetic algorithms and reinforcement learning were used for optimizing dynamic drone networks, demonstrating the overall necessity of adaptive decision-making strategies for UAVs when the environmental conditions and network state change dynamically. It appears that the above studies demonstrate great achievements in temporal modeling, multimodal sensing, feature representation, and UAV reliability. Nonetheless, the evaluation is mostly conducted either using sample data, a windowing approach, predefined scenarios, or fault recognition ability.

### 2.2. Federated Learning for UAV and Aviation Systems

Federated learning is a very promising approach to consider for unmanned aerial vehicles, considering that aerial vehicles naturally create a distributed dataset amid communication constraints, privacy constraints, energy constraints, and computational constraints. Study [21] is a comprehensive study on statistical heterogeneity in federated learning for UAVs and considers non-identical distributions of data among clients as one of the main challenges to the process of collaborative learning. The field of aviation is considered in study [4], where federated learning with imbalanced data is explored, and the researchers find that aviation data is distributed, which requires learning techniques accounting for class distribution differences among participants.

Recent studies have therefore focused on privacy-preserving and communication-efficient aggregation approaches. Study [22] proposes FedShufde for smart UAV delivery in the edge computing environment by combining federated learning with differential privacy and shuffling for protecting UAV-specific privacy while hiding the client–server association. Study [23] has proposed FedWT, in which, instead of the conventional centralized aggregation approach, minimum spanning tree-based weighted aggregation is used in order to increase robustness against unreliable UAV channels and reduce communication overhead in the presence of heterogeneous data. Study [24] has proposed DFUN-KDF, which involves a decentralized approach where UAV models exchange their distilled embeddings instead of entire model parameters.

Centralized, decentralized, and federated intelligence choices have become a popular topic of research in multi-UAV systems. Study [25] compares centralized, decentralized, and federated reinforcement learning approaches for multi-UAV swarms and shows how the coordination framework affects the scalability of the system, the learning process, and the efficiency of learning. Research presented in [26] investigates multi-cell federated learning with wireless packet errors and highlights that unreliable communication in the air affects the collaboration of intelligence and has to be taken into account when designing UAV learning systems. Study [27] proposes a FedGTD-UAVs framework combining federated transfer learning and a detection network for robust small-target recognition in UAV swarms and proves that federated transfer of knowledge can help perform sensing tasks without fully collecting distributed data.

Client heterogeneity has motivated increasingly specialized aggregation mechanisms. Study [28] developed a cost-efficient federated aggregation strategy for UAV swarm networks under non-IID data, explicitly balancing learning performance with the resource constraints of aerial clients. Study [29] addressed heterogeneous few-shot specific-emitter identification for low-altitude drone management by aggregating feature representations across distributed receivers with mismatched label spaces, showing that carefully designed federated representation learning can approach centralized performance even under severe heterogeneity. Study [30] combined federated learning with semantic communication and PPO-based trajectory and resource optimization, demonstrating that model training, communication efficiency, and UAV motion planning can be jointly considered rather than optimized independently.

Federated UAV research has also expanded beyond standard FedAvg toward incentive, security, and hybrid optimization mechanisms. A reward-and-punishment-aware incentive mechanism is developed in [31] for DAG-blockchain-based federated learning in UAV networks to encourage reliable participation in decentralized aggregation. Study [32] proposed a secure hybrid split-federated learning scheme for UAV-assisted wireless networks, combining distributed learning with game-theoretic resource coordination. Study [33] integrated homomorphic encryption and trusted execution environments into edge-federated UAV communication, illustrating that raw-data locality alone may be insufficient when model updates and aggregation infrastructure are also considered sensitive.

Federated intelligence is similarly being incorporated into mission-level UAV coordination. Study [34] used federated multi-agent reinforcement learning for post-disaster victim localization and explicitly examined non-IID experience, intermittent communication, and heterogeneous devices within a mission-oriented UAV framework. These studies collectively demonstrate that UAV federated learning has advanced rapidly in aggregation, communication, heterogeneity handling, privacy, and distributed decision-making. However, their principal tasks are generally communication optimization, classification, object recognition, emitter identification, resource allocation, or reinforcement-learning policy optimization rather than self-supervised flight anomaly detection evaluated on unseen flight trajectories.

### 2.3. Robustness, Privacy, and Security in Federated UAV Systems

The use of federated learning does not eliminate security risks, because exchanged model parameters or intermediate representations can themselves become attack surfaces. Study [35] proposed FedULite to defend UAV-assisted federated networks against model-poisoning attacks through lightweight unsupervised representation learning and adaptive update filtering, demonstrating that robustness must be considered together with computation and communication constraints. A quantum-resilient federated framework is developed in [36] for multi-layer cyber-anomaly detection in UAV systems, extending distributed UAV security toward cryptographic and post-quantum threat considerations. In a separate study, Study [5] combined vision transformers with Byzantine-robust federated learning for cross-modal intrusion detection in UAV swarms, illustrating the need to protect collaborative models against malicious or unreliable participants.

Security research is also moving toward richer representations of UAV cyber-physical behavior. Study [37] introduced Fed-CGIDS-UAV, which represents UAV telemetry as causal graphs and performs federated cross-domain intrusion detection by identifying violations of learned dependencies among navigation, sensing, communication, control, and actuation variables. Study [38] proposed a federated dynamic multi-scale attention network for secure communication between drones and base stations, demonstrating the use of attention mechanisms for distributed detection under network-security constraints. NeSySwarm-IDS is developed in [39], an end-to-end neuro-symbolic intrusion-detection framework that combines differentiable security rules with federated learning and explicit privacy mechanisms for resource-constrained UAV swarms.

Efficient deployment still remains another equally important requirement in parallel. Study [40] created a computationally efficient IDS solution for UAVs, showing that the efficiency and energy constraints have to be taken into account when creating an accurate predictive model. Study [41] presented the idea of privacy-preserving UAV crowdsensing as a problem of trajectory design, incentives, and federated reinforcement learning, which shows that privacy constraints could directly affect planning and the data collection process. Study [42] carried out an extensive investigation of privacy solutions and metrics in the context of federated learning and showed that keeping raw data local is not enough to provide privacy guarantees, and privacy should be evaluated in the context of attacks and protection measures.

These studies are important for interpreting the role of federated learning in the present work. The objective here is not to claim cryptographic or differential-privacy protection; rather, federated training is used to avoid transferring raw flight telemetry to a centralized training repository. The recent security literature makes this distinction necessary because parameter sharing may still expose information and may also be vulnerable to malicious updates. Accordingly, the present study treats raw-data locality as an architectural benefit while leaving formal privacy guarantees and adversarially robust aggregation for future investigation.

### 2.4. Operational Reliability, Deployment, and Research Gap

Recent UAV research increasingly emphasizes that learning performance must be interpreted within a broader operational context. Study [1] proposed dynamic GNN-based multimodal anomaly detection for spatial crowdsourcing drone services, showing that anomalous behavior may emerge from interactions among mobility, service, and network modalities rather than from a single telemetry stream. Study [43] developed a probabilistic battery-health-management approach for inspection drones, demonstrating that reliable UAV operation requires uncertainty-aware health assessment and mission-level decision support. Study [44] empirically investigated how uncertainty can translate into unsafe UAV behavior, reinforcing the need to connect model uncertainty and prediction errors with safety-relevant system outcomes.

Deployment realism also motivates simulation and communication studies. Study [45] proposed an open-source UAV digital-twin framework for remote-sensing operations, illustrating how digital twins can provide reproducible environments for evaluating aerial-system behavior before physical deployment. Study [46] investigated machine-learning-driven clustering and routing for UAV networks in 6G environments, showing that distributed aerial intelligence must coexist with latency, throughput, mobility, and network-topology constraints. Study [47] reviewed UAV-swarm research, challenges, and future directions and identified communication, coordination, scalability, autonomy, and reliability as persistent barriers to large-scale deployment.

Taken together, the literature shows strong progress in three dimensions. First, UAV anomaly and fault detection has evolved toward temporal, graph-based, multimodal, and uncertainty-aware representations. Second, federated UAV research has developed sophisticated solutions for non-IID data, decentralized aggregation, communication efficiency, privacy, and adversarial robustness. Third, deployment-oriented studies increasingly recognize that mission safety cannot be inferred from a single predictive metric. Nevertheless, a specific methodological gap remains at the intersection of these areas.

The current work distinguishes itself through an approach that incorporates self-supervised telemetry forecasting, federated optimization, and strict flight-disjoint evaluation in one anomaly detection pipeline for UAVs. Windowed evaluation of time series raises special problems if adjacent windows contain a large amount of overlapping data, because highly similar samples coming from the same flight might show up on both sides of a randomized train/test split. Furthermore, although many studies in anomaly detection concentrate on discrimination measures such as AUC, accuracy, F1-Score, etc., an operational warning system should be evaluated for its ability to detect complete failed flights, first alarm delay, and rate of false alarms during normal operation.

In order to solve these problems, the current study learns normal UAV dynamics using self-supervised next-step forecasting while keeping telemetry data in its raw form for federated learning. Complete flights are separated into disjoint folds before creating windows, normalization, calibration, or thresholding in order to test for transfer to completely unseen flights. Furthermore, in addition to measures of discrimination like AUC and average precision, the current study also analyzes the probability of detecting a flight, the time-to-detection, and false alarms per hour in order to distinguish between score-level and alarm-level properties of the model. In this way, the current study will independently evaluate the calibration procedure, local vs. global thresholding, possible leakage of variables from the autopilot, and alarm persistence in order to learn how post-model design choices impact operational performance. Table 1 summarizes the main research directions reviewed above and highlights the limitations most directly related to the hypothesis investigated in this study.

## 3. Methodology

This section describes the experimental methodology used to evaluate federated forecasting for UAV anomaly detection. The framework consists of five main stages: flight-level data preparation and partitioning, self-supervised next-step forecasting, federated model optimization, anomaly score calibration and alarm generation, and flight-level statistical evaluation. Particular attention is given to preventing information leakage between training and held-out flights and to separating anomaly discrimination from operational alarm performance.

**Figure 1:**
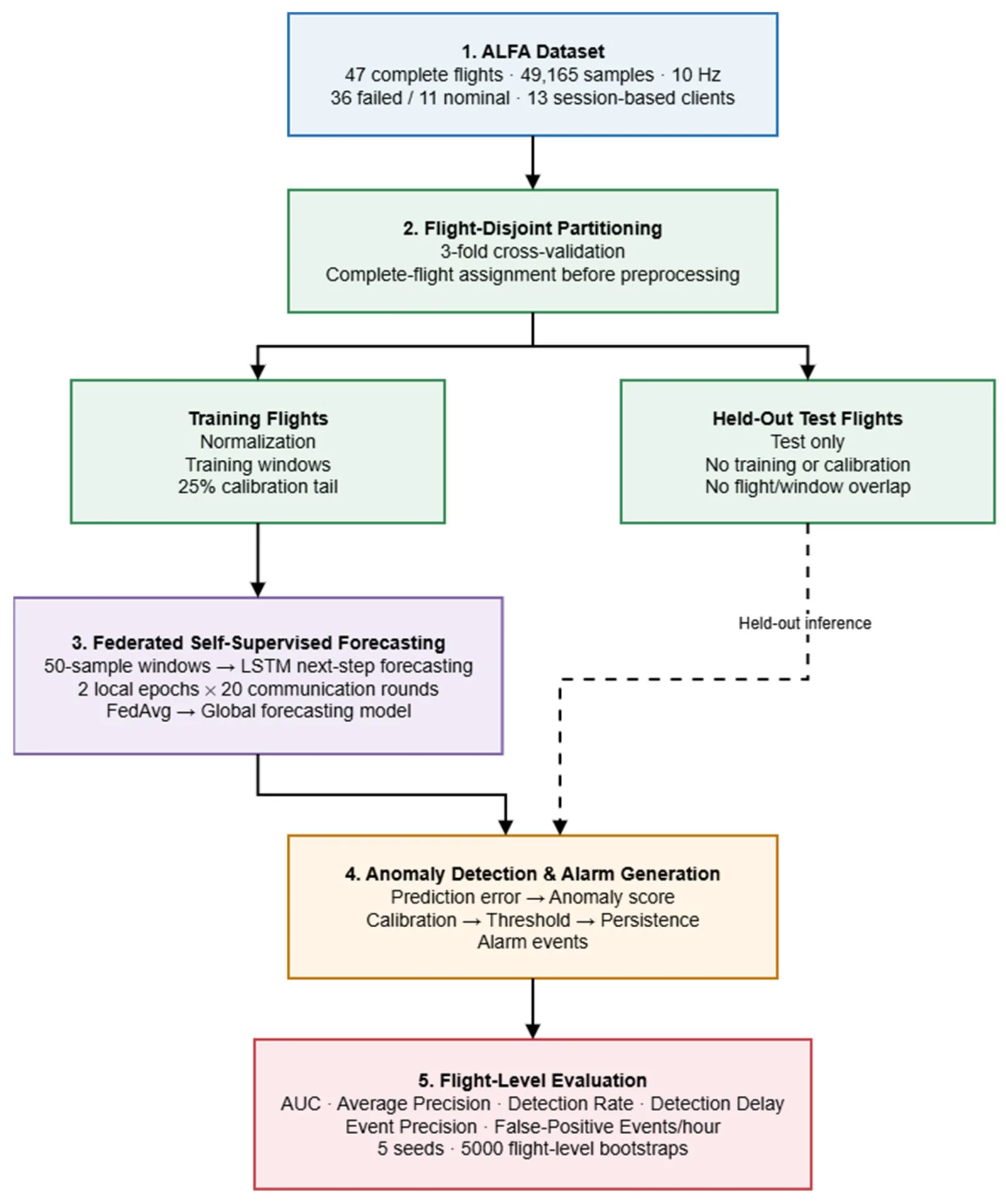
Overview of the proposed federated flight anomaly detection framework and flight-disjoint experimental workflow.

Figure 1 provides an overview of the complete methodological framework and experimental workflow used in this study. The workflow begins with the ALFA dataset and strict flight-disjoint partitioning, in which complete flights are assigned to training or held-out test sets before preprocessing and window generation. Training flights are used for self-supervised federated forecasting and calibration, whereas held-out flights are used exclusively for anomaly detection and evaluation. The resulting forecasting errors are converted into anomaly scores and subsequently processed through calibration, thresholding, and temporal persistence to generate alarm events. Finally, performance is assessed using both discrimination-oriented and operational metrics, including AUC, average precision, flight-level detection rate, detection delay, event precision, and false-positive events per hour.

### 3.1. Dataset, Preprocessing, and Flight-Disjoint Protocol

This study uses the publicly available AirLab Failure and Anomaly (ALFA) dataset introduced in [8] for UAV fault detection and anomaly detection research. The processed ALFA collection contains 47 autonomous flight sequences with annotated failure types and failure times. After synchronization and preprocessing according to the protocol described below, the subset used in the present experiments comprises 49,165 telemetry samples corresponding to approximately 81.9 min of operation. Thirty-six flights contain an injected failure, and 11 are nominal. The failures comprise 23 engine, eight aileron, three rudder, and two elevator cases; the mixed rudder–aileron case is assigned to the first matching isolation category. ALFA was selected because its data organization is well suited to the evaluation objective of this study: telemetry is available as complete temporal flight trajectories with annotated failure occurrences, allowing entire flights to be withheld before preprocessing and enabling both anomaly score discrimination and post-onset operational measures such as flight-level detection and detection delay to be evaluated without fragmenting individual missions. The subset also contains multiple control-related failure categories and nominal flights, permitting evaluation across different failure conditions together with false-alarm measurement during failure-free operation. Nevertheless, the 47 flights should not be interpreted as statistically representative of the full diversity of UAV platforms, missions, environments, or failure mechanisms encountered in operational fleets. The dataset is therefore used as a controlled benchmark for flight- and session-level generalization rather than as a population-representative sample of UAV operations. MAVROS topics are resampled to a common 10 Hz grid and joined into one table per flight. The retained signals include commanded and measured attitude, airspeed and velocity, inertial measurements, local velocity, throttle, altitude, battery, wind estimates, and actuator outputs. Command–measurement residuals are derived where paired channels are available. Four autopilot-computed tracking-error variables are excluded from the primary 52-channel feature set because they can act as near-direct proxies for a control failure; their inclusion is evaluated only as an ablation.

All preprocessing operations are performed independently within each outer cross-validation fold. Feature-normalization parameters are estimated exclusively from failure-free samples belonging to the training flights and are subsequently applied unchanged to the calibration and held-out test flights. The telemetry channels are standardized using client-specific z-score normalization. For feature j of client k, the standardized value is computed using Equation (1):

(1) $${\tilde{x}}_{k,t,j}=\frac{x_{k,t,j}-\mu_{k,j}}{\sigma_{k,j}+\varepsilon },\;\;\varepsilon =10^{-6},$$

where $x_{k,t,j}$ denotes the original value of feature j at time t, and $\mu_{k,j}$ and $\sigma_{k,j}$ are the mean and standard deviation estimated exclusively from the corresponding client’s failure-free training samples. A global estimate computed from failure-free samples in the outer training fold is used only as a fallback for cold-start clients without an eligible local training flight.

Following feature standardization, the synchronized telemetry streams are segmented into windows of length *L* = 50 samples, corresponding to 5 s at the 10 Hz sampling frequency, with a stride of one sample. Each input window is used to predict the immediately following 52-dimensional telemetry vector. Flight assignment to the training and test partitions is completed before normalization or window selection, preventing information from held-out flights from entering the preprocessing pipeline. The dataset characteristics and main evaluation settings are summarized in Table 2.

Flights recorded within the same date-hour block are assigned to the same federated client, which serves as a proxy for an operational recording session. This grouping maps the 47 individual flights to 13 heterogeneous clients with different amounts of local data. A client may contribute different complete flights to the training and test folds, but no individual flight, sample, or window derived from that flight is divided across a fold boundary. These clients should therefore be interpreted as 13 session-based partitions, not as 13 distinct physical UAVs.

Three-fold cross-validation is assigned within client and failure-presence strata. No sample or overlapping window from a test flight can contribute to model training, feature normalization, score calibration, or threshold estimation. This separation is particularly important because adjacent 50-sample windows generated with a stride of one share 49 samples. The resulting protocol evaluates generalization to unseen flights rather than memorization of flight-specific temporal patterns.

To further distinguish unseen-flight generalization from unseen-session generalization, an additional session-disjoint evaluation was conducted. In this experiment, the 13 date-hour session proxies were treated as indivisible groups during cross-validation, such that all flights belonging to a given session were assigned exclusively to either the training or test partition within each fold. Consequently, no session contributed flights to both sides of a fold boundary. Held-out sessions were treated as cold-start clients; therefore, their telemetry was not used to estimate client-specific normalization statistics, calibration parameters, or alarm thresholds. Instead, normalization and global threshold estimation relied exclusively on the corresponding training sessions. All remaining model, training, and alarm-processing settings were kept consistent with the conservative FedAvg configuration used in the main flight-disjoint analysis. The experiment was repeated with five random seeds.

### 3.2. Self-Supervised Forecasting Model

Following the recurrent forecasting formulation commonly used for temporal anomaly detection [6,18], the multivariate UAV telemetry is modeled as a next-step prediction problem.

Let $x_{t}\in R^{d}$ denote the multivariate telemetry vector observed at time step t, where *d* = 52. For a forecasting window of length L, the input sequence is defined in Equation (2):

(2) $$X_{t}=\left[x_{t-L+1},x_{t-L+2},\ldots ,x_{t}\right]$$

The LSTM forecaster parameterized by θ predicts the immediately following telemetry vector according to Equation (3):

(3) $${\hat{x}}_{t+1}=f_{\theta }\left(X_{t}\right)$$

The task is self-supervised because the prediction target $x_{t+1}$ is obtained directly from the telemetry sequence and no failure label is used as a prediction target during model optimization. Training windows are restricted to failure-free portions of the outer training flights.

At inference, the disagreement between the predicted and measured telemetry vectors is converted into a scalar anomaly score using mean squared next-step forecasting error defined in (4):

(4) $$s_{t+1}=\frac{1}{d}\sum_{j=1}^{d}{\left(x_{t+1,j}-{\hat{x}}_{t+1,j}\right)}^{2},\;\;d=52$$

Larger values of $s_{t+1}$ indicate greater deviation from the failure-free temporal dynamics learned during training. The forecasting network contains one unidirectional LSTM layer with 64 hidden units. Its input dimension is 52, and its final hidden representation is passed to a fully connected linear layer with 52 outputs. No output activation is applied because the network performs multivariate regression, and the single-layer LSTM uses zero dropout.

To assess whether the observed findings depend specifically on the LSTM forecasting backbone, an additional controlled experiment was conducted using a gated recurrent unit (GRU) network. The GRU used the same single recurrent layer with 64 hidden units and the same 52-dimensional forecasting output as the LSTM. All other experimental conditions were held fixed, including the input representation, optimizer, training budget, FedAvg configuration with 20 communication rounds and full client participation, five repeated seeds, three-fold flight-disjoint cross-validation, and downstream anomaly score processing. Both models were evaluated using rolling calibration, a global $Q_{0.99}$ threshold, and 0.5 s temporal persistence. This design isolates the effect of the recurrent forecasting backbone while preserving the remainder of the federated anomaly detection pipeline.

For client k, the mean squared forecasting loss minimized during local training is defined in Equation (5):

(5) $$L_{k}\left(\theta \right)=\frac{1}{N_{k}}\sum_{i=1}^{N_{k}}{\left\|x_{t+1}^{\left(i\right)}-f_{\theta }\;\left(X_{t}^{\left(i\right)}\right)\right\|}_{2}^{2}$$

where $N_{k}$ denotes the number of eligible training windows available to client k. Optimization uses Adam with a learning rate of $10^{-3}$ and mini-batches of 256 windows. Each client performs two local epochs per communication round. The model architecture and main training hyperparameters are summarized in Table 3.

### 3.3. Federated Learning and Baseline Configuration

Let K denote the number of active clients in a communication round. At round r, client k initializes its local model using the current global parameters $\theta^{\left(r\right)}$ and performs two local training epochs over its eligible failure-free training windows. Following the Federated Averaging (FedAvg) formulation introduced in [48], the locally updated parameters are aggregated according to the number of local training windows, as shown in Equation (6):

(6) $$\theta^{\left(r+1\right)}=\sum_{k=1}^{K}\frac{n_{k}}{\sum_{j=1}^{K}n_{j}}\theta_{k}^{\left(r+1\right)}$$

where $n_{k}$ denotes the number of training windows belonging to client k, $\theta_{k}^{\left(r+1\right)}$ denotes its locally optimized parameters, and $\theta^{\left(r+1\right)}$ is the resulting global model. Clients with larger effective local training sets consequently contribute proportionally more to the global update.

FedProx extends the federated optimization objective with a proximal regularization term to account for client heterogeneity, following [49], as defined in Equation (7):

(7) $$L_{k}^{prox}\left(\theta \right)=L_{k}\left(\theta \right)+\frac{\mu }{2}{\left\|\theta -\theta^{\left(r\right)}\right\|}_{2}^{2},\;\;\mu =0.01$$

Personalized FedAvg takes the learned FedAvg global model and fine-tunes a private copy for each client for five additional epochs using only that client’s eligible failure-free training data. The personalized parameters remain local and are not aggregated back into the global model. A local-only baseline independently trains one forecasting model per client without parameter exchange. The centralized baseline pools all eligible failure-free training windows in the corresponding outer fold and uses the same architecture, optimizer-restart schedule, number of rounds, and epochs-per-round budget.

For every training flight, the final 25% of its eligible windows are reserved as a calibration tail. These windows are excluded from gradient-based optimization and used only for anomaly score calibration and threshold estimation. No telemetry window from a held-out test flight is used for model fitting, feature normalization, calibration, or threshold selection. A runtime assertion terminates an experiment if any train-test flight overlap is detected.

To evaluate the sensitivity of federated training to intermittent client availability, an additional controlled partial-participation experiment was conducted using FedAvg. In each communication round, either 100%, 75%, or 50% of the eligible training clients participated in local optimization and subsequent aggregation. For the partial-participation configurations, the participating clients were randomly selected at each communication round according to the experiment seed. All other components of the experimental protocol, including the flight-disjoint folds, model architecture, 20 communication rounds, two local epochs per round, preprocessing, calibration, and evaluation procedure, were kept unchanged. This experiment isolates the effect of reduced client participation while avoiding confounding changes to the underlying forecasting and evaluation pipeline. The complete federated training and flight-level evaluation procedure is summarized in Algorithm 1.
**Algorithm 1:** Federated forecasting and flight-level evaluation1. Assign complete flights to three disjoint folds within client&failure strata. 2. For each outer fold, compute normalization statistics using normal samples from training flights only. 3. Construct length-L next-step forecasting windows; reserve the final 25% of eligible normal training windows for threshold calibration. 4. Initialize global parameters $\theta^{\left(0\right)}$. 5. For each round *r* = 0, …, R−1: distribute $\theta^{\left(r\right)}$, train each active client locally, and aggregate updates using Equation (6). 6. Score every held-out flight using Equation (4), then apply robust calibration and a threshold learned without test data. 7. Apply the selected persistence rule, record the first post-onset alarm, and merge consecutive normal alarms into events. 8. Aggregate flight-level metrics over five seeds and compute 95% confidence intervals by stratified flight bootstrap.

### 3.4. Anomaly Score Calibration and Alarm Logic

Raw prediction-error magnitudes vary between flights because of differences in wind, trim, payload, sensor offsets, and other operating conditions. Three score-calibration configurations are evaluated: robust rolling calibration, warm-up calibration, and no calibration.

The rolling configuration uses a trailing baseline spanning *W* = 30 s and delayed by *D* = 5 s so that a developing anomaly does not immediately contaminate its own reference. Let $\ell =\left\lceil D/\Delta t\right\rceil$ and $w=\left\lceil W/\Delta t\right\rceil$. The implementation first shifts the score stream, $b_{t}=s_{t-\ell }$, and computes rolling medians according to Equation (8).

(8) $$m_{t}=\mathrm{median}\left\{b_{u}:u=t-w+1,\ldots ,t\right\},\;q_{t}=\mathrm{median}\left\{\left|b_{u}-m_{u}\right|:u=t-w+1,\ldots ,t\right\}$$

The calibrated score is then obtained using Equation (9).

(9) $$z_{t}=\frac{s_{t}-m_{t}}{1.4826\;q_{t}+10^{-9}}$$

The equation reproduces the implemented robust rolling transformation, including the Gaussian-consistency factor 1.4826. The rolling calculations require at least $\max\left(10,\left\lfloor w/4\right\rfloor \right)$ available observations; unavailable leading values are set to zero, and the first W + D = 35 s are excluded from false-alarm exposure. Warm-up calibration instead computes a fixed median and median absolute deviation from the opening 20 s and applies the same 1.4826 scaling. In the uncalibrated condition, $z_{t}=s_{t}$.

Alarm thresholds are estimated exclusively from failure-free calibration windows. For client k, the client-specific threshold is determined according to Equation (10),

(10) $$\tau_{k}=Q_{0.99}\;\left(S_{k}^{cal}\right)$$

where $S_{k}^{cal}$ is the set of eligible calibrated scores associated with client k. The global threshold is the median of the available client thresholds, as defined in Equation (11):

(11) $$\tau_{global}=\mathrm{median}\left(\tau_{1},\tau_{2},\ldots ,\tau_{K}\right)$$

To reject isolated score spikes, a temporal-persistence requirement may be imposed. For persistence duration p and sampling interval Δt, the required run length is $m_{p}=\max\left(1,\left\lceil p/\Delta t\right\rceil \right)$. The alarm indicator is defined in Equation (12),

(12) $$a_{t}=I\left[\overset{m_{p}-1}{\underset{i=0}{⋂}}\left(z_{t-i}>\tau \right)\right]=\left\{\begin{array}{cc} 1, & z_{t-m_{p}+1}>\tau ,\ldots ,z_{t}>\tau ,\\ 0, & \mathrm{otherwise}. \end{array}\right.$$

When *p* = 0, $m_{p}$ = 1, and a single threshold exceedance activates the alarm. Detection time is the time at which the condition is first completed after the annotated failure onset. Persistence, therefore, cannot reduce false alarms without incurring its corresponding latency cost. Consecutive active alarm samples during nominal operation are merged into one false-alarm event.

### 3.5. Evaluation Metrics and Statistical Analysis

Performance is evaluated using both discrimination-oriented and operational metrics. Per-flight AUC and average precision quantify anomaly score ranking on failure-containing flights. Flight-level detection rate, detection delay, normal-window false-alarm rate, and false-alarm events per hour characterize operational alarm behavior.

Let $N_{failed}$ be the number of evaluated failure-containing flights and $N_{detected}$ the number for which at least one valid post-onset alarm is generated. The flight-level detection rate is calculated according to Equation (13)

(13) $$Detection\;Rate=\frac{N_{detected}}{N_{failed}}$$

For a detected failed flight i, detection delay is calculated using Equation (14)

(14) $$D_{i}=t_{i}^{alarm}-t_{i}^{onset}$$

where $t_{i}^{onset}$ is the annotated failure-onset time and $t_{i}^{alarm}$ is the time at which the first valid post-onset alarm is completed. The reported delay is $\mathrm{median}\left\{D_{i}:i\in D\right\}$, where *D* is the set of detected failed flights. Detection delays are computed relative to the failure-onset timestamps provided by the ALFA annotations. Because the temporal uncertainty of these onset annotations is not independently quantified in the dataset, small sub-second differences in reported detection delay should not be interpreted as evidence of practically meaningful timing differences between configurations.

False-alarm burden is expressed as the number of distinct false-alarm events per hour of eligible nominal exposure according to Equation (15):

(15) $$False\;Alarms/Hour=\frac{N_{FA}}{T_{normal}/3600}$$

where $N_{FA}$ is the number of false-alarm events after merging consecutive active nominal alarm samples and $T_{normal}$ is the eligible nominal exposure in seconds. The normal-window false-alarm rate is additionally calculated using Equation (16).

(16) $$FAR=\frac{N_{alarm,normal}}{N_{normal}}$$

To further characterize operational alarm quality, event-level precision is calculated in addition to the false-alarm rate. An alarm event is defined as a contiguous run of active alarm samples. For each failure-containing flight, only the first valid alarm event completed after the annotated failure onset is counted as a true-positive event; subsequent alarm events associated with the same failure do not increase the true-positive count. Alarm events occurring during eligible nominal exposure, including the pre-onset portion of failure-containing flights, are counted as false-positive events. Event-level precision is calculated using Equation (17).

(17) $${Precision}_{event}=\frac{N_{TP,event}}{N_{TP,event}+N_{FP,event}}$$

Absolute false-positive event counts and false-positive events per hour are reported together to characterize both the number and exposure-normalized frequency of erroneous alarms.

To examine more conservative operating regimes, the rolling-calibrated FedAvg detector is additionally evaluated using calibration quantiles of 0.99, 0.995, 0.999, and 0.9995 and persistence durations of 0, 0.1, 0.3, 0.5, 1, 2, and 3 s for both client-specific and global thresholds, resulting in 56 operating configurations. All thresholds are estimated exclusively from the normal calibration tails of the outer training folds, without using test scores for threshold selection. For interpretability, operating points satisfying analytical targets of ≤10, ≤5, and ≤1 false-positive events per hour are examined. These values are used only as conservative analytical targets and are not presented as standardized deployment-acceptance criteria.

All headline experiments are repeated with five random seeds. Statistical uncertainty is estimated using 5000 stratified bootstrap repetitions. Complete flights, rather than individual telemetry windows, are the resampling unit, and all five seed-specific observations associated with a sampled flight are retained together. Flight-level resampling preserves within-flight temporal dependence and avoids artificially narrow confidence intervals that would arise if strongly overlapping windows were treated as independent observations.

## 4. Results

The experimental results are presented in three stages. First, the federated forecasting variants are compared with local-only and centralized training under the flight-disjoint protocol. Second, calibration strategy, threshold scope, and the inclusion of autopilot-derived tracking-error channels are examined through controlled ablations. Finally, the interaction among detection probability, detection delay, and false-alarm burden is analyzed across different threshold and persistence configurations. Unless otherwise stated, all reported results are aggregated across three flight-disjoint folds and five random seeds.

### 4.1. Federated Versus Local and Centralized Learning

We first compare local-only training, FedAvg, FedProx, personalized FedAvg, and centralized training using the same flight-disjoint folds and optimization budget. The purpose of this experiment is to determine whether federated parameter aggregation can recover the performance lost when each client is restricted to its own local telemetry.

**Figure 2:**
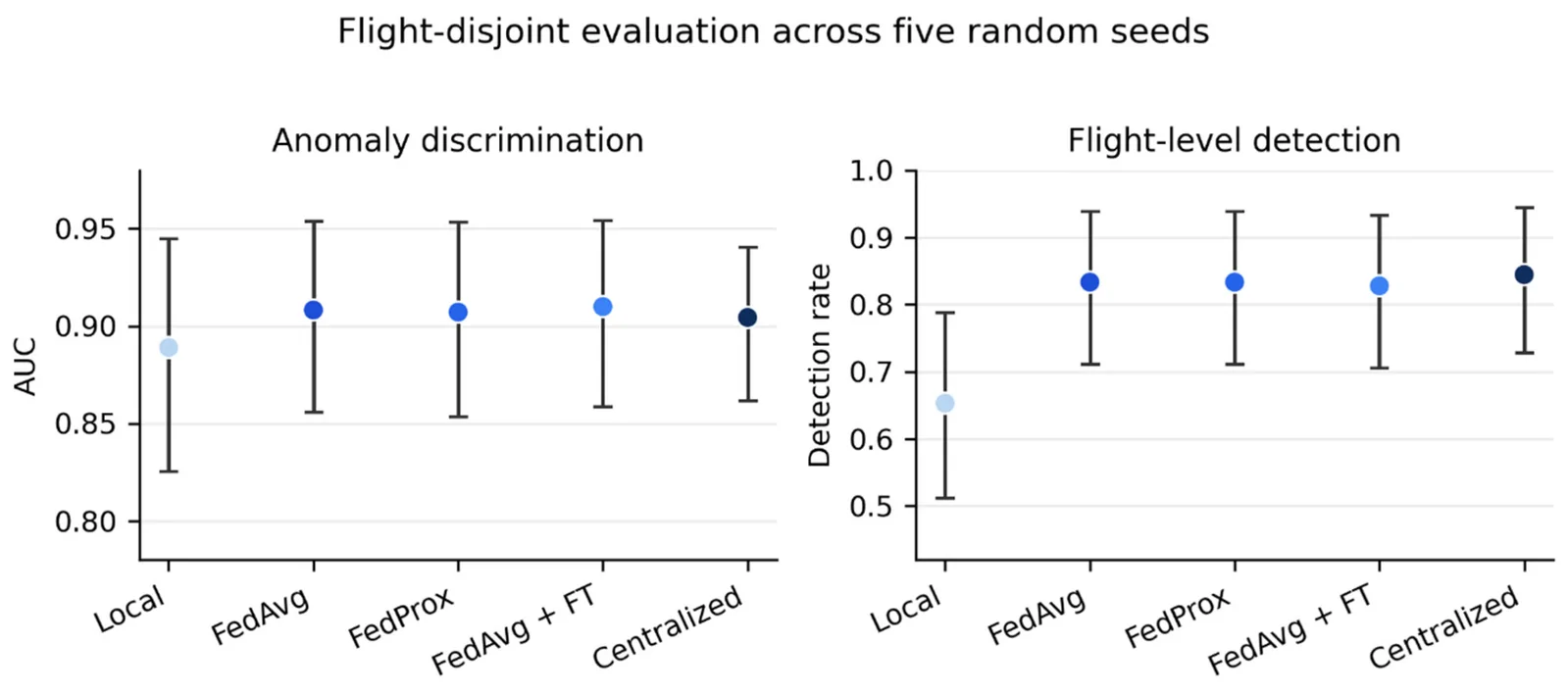
AUC and flight-level detection rate under flight-disjoint evaluation. Error bars are 95% confidence intervals from 5000 flight-bootstrap repetitions.

Figure 2 shows that the three federated variants remain close to centralized training in anomaly discrimination while achieving markedly higher point estimates of flight-level detection than local-only training. FedAvg achieved an AUC of 0.908, compared with 0.904 for centralized training and 0.889 for local training. A similar pattern is observed for average precision, although the separation is more pronounced: FedAvg and FedProx both achieved an AP of 0.816 and personalized FedAvg reached 0.818, whereas centralized training obtained 0.762. This difference suggests that comparable ROC-based discrimination does not necessarily imply equivalent precision–recall behavior under the class distribution encountered in the held-out flights. FedAvg and FedProx both detected 83.3% of failed flights, personalized FedAvg detected 82.8%, and centralized training detected 84.4%. These flight-level detection estimates are based on the 36 failure-containing flights and are therefore subject to substantial sampling uncertainty. As shown by the 95% flight-bootstrap confidence intervals in Figure 2, the intervals overlap substantially; consequently, the small differences between FedAvg and centralized training should not be interpreted as evidence of a meaningful difference in detection performance. Rather, the results support the more limited conclusion that their observed detection performance is comparable within the uncertainty of the present dataset.

Table 4 reveals why AUC alone is insufficient for method selection. Local training produced the fewest alarm events but missed approximately one-third of failed flights and had the longest delay. FedAvg increased the flight-level detection rate from 65.3% to 83.3% and reduced the median detection delay from 2.67 s to 0.47 s relative to local-only training. This gain, however, was accompanied by a substantial increase in alarm burden, from 43.1 to 258.5 false-alarm events per hour under the default warm-up/personal-threshold operating point. At the evaluated proximal coefficient of μ = 0.01, FedProx did not provide a meaningful advantage over the simpler FedAvg update, yielding nearly identical AUC, detection rate, detection delay, and false-alarm burden. Because μ was not systematically tuned in the present study, this result should not be interpreted as evidence that proximal regularization is ineffective more generally. Personalized fine-tuning slightly increased AUC from 0.908 to 0.910 and reduced the false-alarm burden from 258.5 to 193.2 events per hour; however, the flight-level detection rate slightly decreased from 83.3% to 82.8%, while the median detection delay increased from 0.47 s to 1.45 s. Given the absence of a clear overall benefit from FedProx at μ = 0.01 or from the evaluated personalization configuration, FedAvg was retained as the simplest federated backbone for the subsequent calibration and alarm-policy experiments.

An additional controlled experiment was conducted to quantify the effect of partial client participation on FedAvg. Table 5 reports the results for 100%, 75%, and 50% client participation. Reducing participation from 100% to 75% produced the same AUC (0.9123) and flight-level detection rate (82.78%), while AP changed from 0.8587 to 0.8565, median detection delay increased from 3.38 to 4.17 s, and the false-positive event rate increased from 8.17 to 8.49 events/h. At 50% participation, the detector achieved an AUC of 0.9117, an AP of 0.8565, a flight-level detection rate of 83.33%, a median detection delay of 3.48 s, and 8.17 false-positive events/h.

The forecasting backbone was additionally evaluated by replacing the LSTM with a GRU while keeping the remaining experimental configuration unchanged. Table 6 presents the resulting comparison. The LSTM achieved an AUC of 0.9123 and an AP of 0.8587, compared with 0.9108 and 0.8473 for the GRU, respectively. Flight-level detection was 82.78% for the LSTM and 81.11% for the GRU, while the corresponding median detection delays were 3.38 s and 4.50 s. Both architectures produced the same false-positive event rate of 8.17 events/h. The 95% flight-bootstrap AUC intervals were [0.860, 0.957] for the LSTM and [0.859, 0.955] for the GRU, while the corresponding detection intervals were [0.706, 0.928] and [0.694, 0.917].

### 4.2. Calibration and Feature Ablations

Because FedAvg achieved competitive discrimination and detection without the additional proximal or personalization mechanisms, it is used as the common backbone for the remaining ablation experiments. Starting from the warm-up-calibrated, client-specific-threshold configuration without autopilot-derived tracking-error channels, we vary one design factor at a time to isolate the effects of score calibration, threshold scope, and potentially leaky features.

**Figure 3:**
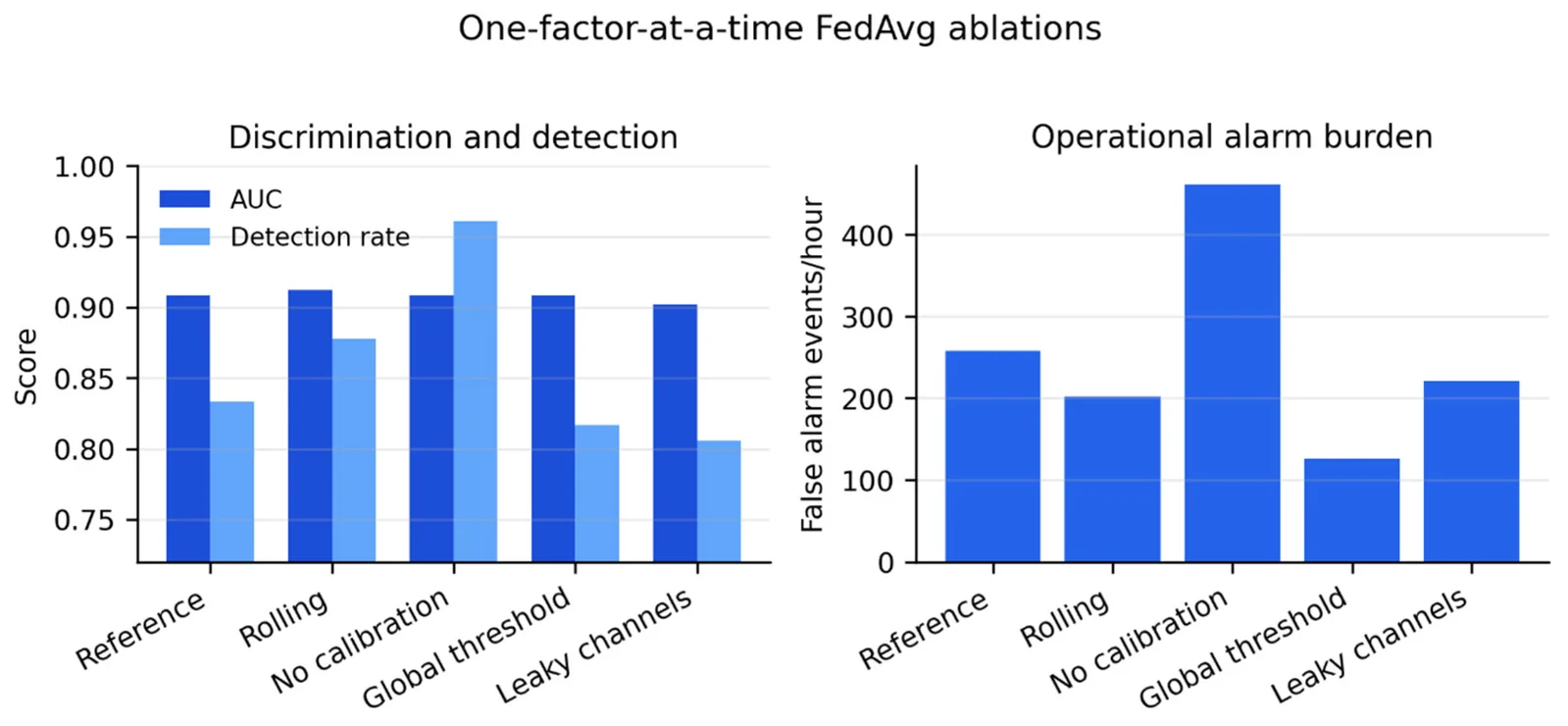
Effect of score calibration, threshold scope, and autopilot-derived tracking-error channels on FedAvg.

As illustrated in Figure 3, rolling calibration provided the best observed discrimination–detection combination, reaching 0.912 AUC and 87.8% detection while reducing alarm events from 258.5 to 202.1 per hour relative to the warm-up reference. Removing calibration increased detection to 96.1%, but this apparent improvement was accompanied by 461.5 false alarms per hour. A global threshold reduced the warm-up alarm burden to 126.5 events per hour, although detection fell to 81.7% and median delay rose to 1.75 s. Including the four potentially leaky autopilot-derived tracking-error channels did not improve the headline result: AUC fell to 0.902 and detection to 80.6%. The primary result, therefore, does not depend on those near-proxy channels. Taken together, these results identify rolling calibration with the non-leaky feature set as the most suitable score-processing configuration for the subsequent operating-point analysis.

Table 7 demonstrates that calibration and threshold selection primarily alter the operational point rather than the underlying rank ordering of anomaly scores. Across the evaluated configurations, AUC remains within a narrow range of 0.902–0.912, whereas detection varies from 80.6% to 96.1% and false-alarm burden ranges from 126.5 to 461.5 events per hour. The uncalibrated setting is a particularly clear example: its 96.1% detection rate would appear favorable if alarm burden were omitted, but it also produces the highest false-alarm rate in the ablation study. These results reinforce the distinction between discrimination quality and operational threshold behavior.

### 4.3. Detection-Delay-False-Alarm Operating Trade-Off

Having selected rolling calibration and the non-leaky feature set, we next examine how threshold scope and temporal persistence jointly determine the operational behavior of the detector. All operating points in this experiment are generated from the same rolling-calibrated FedAvg score streams; therefore, differences in detection, delay, and false-alarm burden arise from alarm-policy choices rather than from retraining the forecasting model.

**Figure 4:**
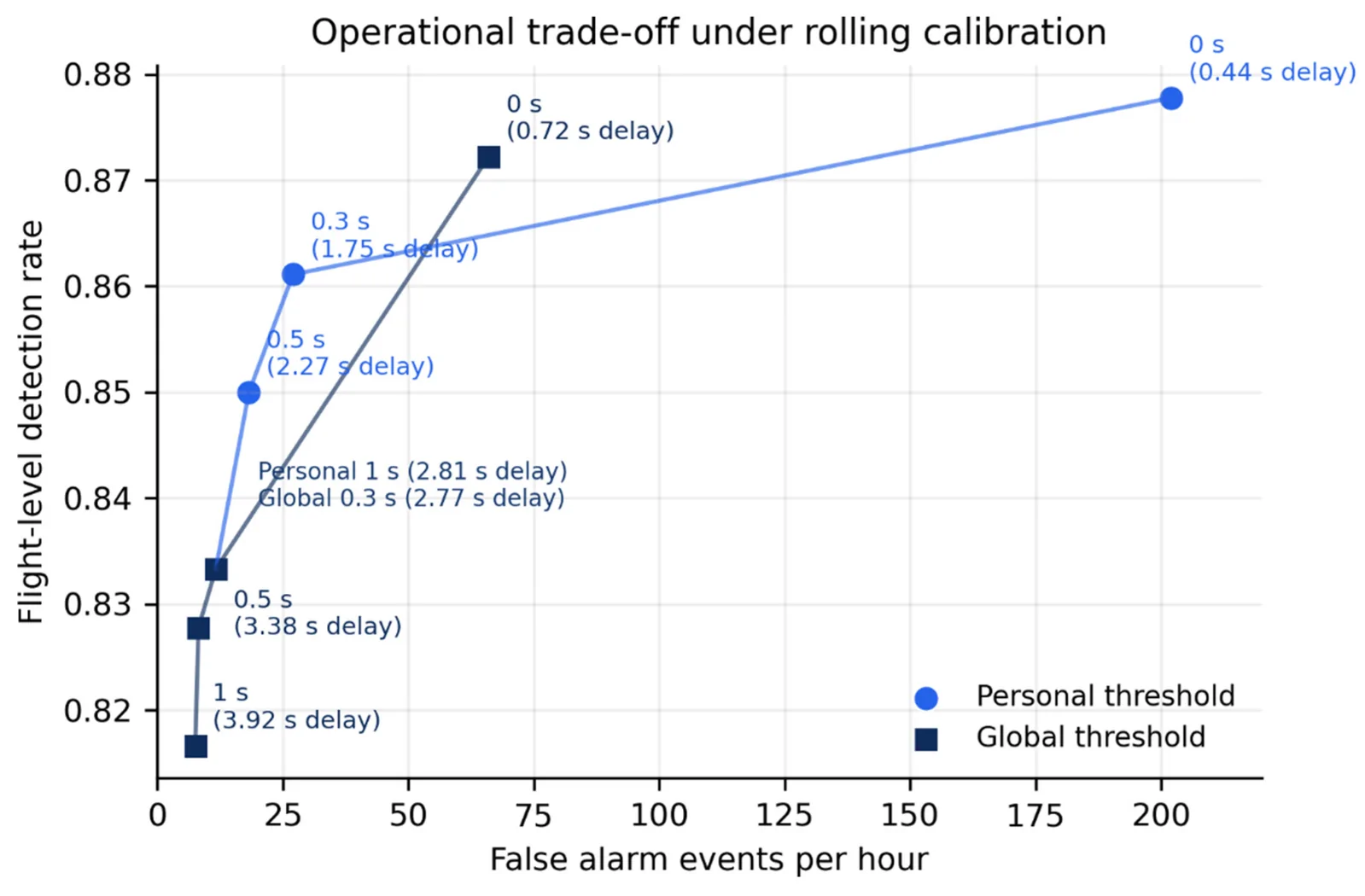
Flight-level detection versus false alarm events per hour for personal and global thresholds. Labels show persistence duration and median detection delay.

Figure 4 presents the final operating-point sweep using identical rolling-calibrated FedAvg score streams. Without persistence, replacing personal thresholds with a global threshold reduced false alarms from 202.1 to 66.0 events per hour, while detection decreased only from 87.8% to 87.2% and median delay increased from 0.44 to 0.72 s. This configuration provides the most favorable low-latency trade-off among the evaluated operating points, retaining 87.2% detection with a median delay of 0.72 s while reducing the alarm burden by approximately two-thirds relative to the personal-threshold configuration without persistence. When a lower alarm burden is required, a second practically attractive trade-off is obtained using a personal threshold with 0.3 s persistence. This configuration retained 86.1% detection and reduced the alarm burden to 27.0 events per hour, although the median detection delay increased to 1.75 s.

Longer persistence produced progressively fewer false alarms but diminishing operational returns. Personal-threshold persistence of 0.5 s yielded an 85.0% detection, a 2.27 s delay, and 18.2 alarms per hour. A global threshold with 1.0 s persistence reached 7.5 alarms per hour, but detection fell to 81.7%, and delay increased to 3.92 s. Table 8 summarizes the representative operating points highlighted in Figure 4, illustrating how threshold scope and temporal persistence jointly affect flight-level detection, detection latency, and false-alarm burden.

The operating-point analysis further demonstrates the trade-off between false-alarm suppression and detection performance. As summarized in Table 9, progressively more conservative configurations substantially reduce the false-alarm burden and improve event-level precision, but at the cost of lower failed-flight detection and increased detection latency. The previously reported global-threshold configuration at $Q_{0.99}$ without persistence achieved 87.2% flight-level detection with a median delay of 0.72 s, but generated 210 false-positive events over the pooled evaluation exposure, corresponding to 66.0 events/h and an event precision of 0.428.

The TP and FP event counts in Table 9 are pooled across 235 flight-seed evaluations (47 flights × 5 random seeds) and therefore should not be interpreted as observations from 235 independent physical flights. The eligible nominal exposure totals approximately 3.18 h across the five seed-specific evaluations. Consequently, low false-alarm rates should be interpreted together with the corresponding absolute event counts. Among configurations satisfying each analytical false-alarm target, the reported operating point was selected by prioritizing flight-level detection rate, followed by median detection delay and event precision.

A substantially more conservative operating point was obtained using a global $Q_{0.99}$ threshold with 0.5 s persistence. This configuration reduced the false-alarm burden to 26 events, or 8.17 events/h, while retaining 82.8% flight-level detection and achieving an event precision of 0.851; however, the median detection delay increased to 3.38 s. Tightening the analytical target to ≤5 events/h required a global $Q_{0.995}$ threshold with 2.0 s persistence, yielding 14 false-positive events (4.40 events/h) and an event precision of 0.903, but reducing detection to 72.2% and increasing the median delay to 6.05 s. No evaluated configuration achieved ≤1 false-positive event/h. The minimum observed false-alarm rate was 4.09 events/h and was associated with further degradation in detection performance and latency. These results demonstrate that reducing the false-alarm burden substantially improves event precision, but only through a trade-off involving lower failed-flight detection and increased detection latency.

**Figure 5:**
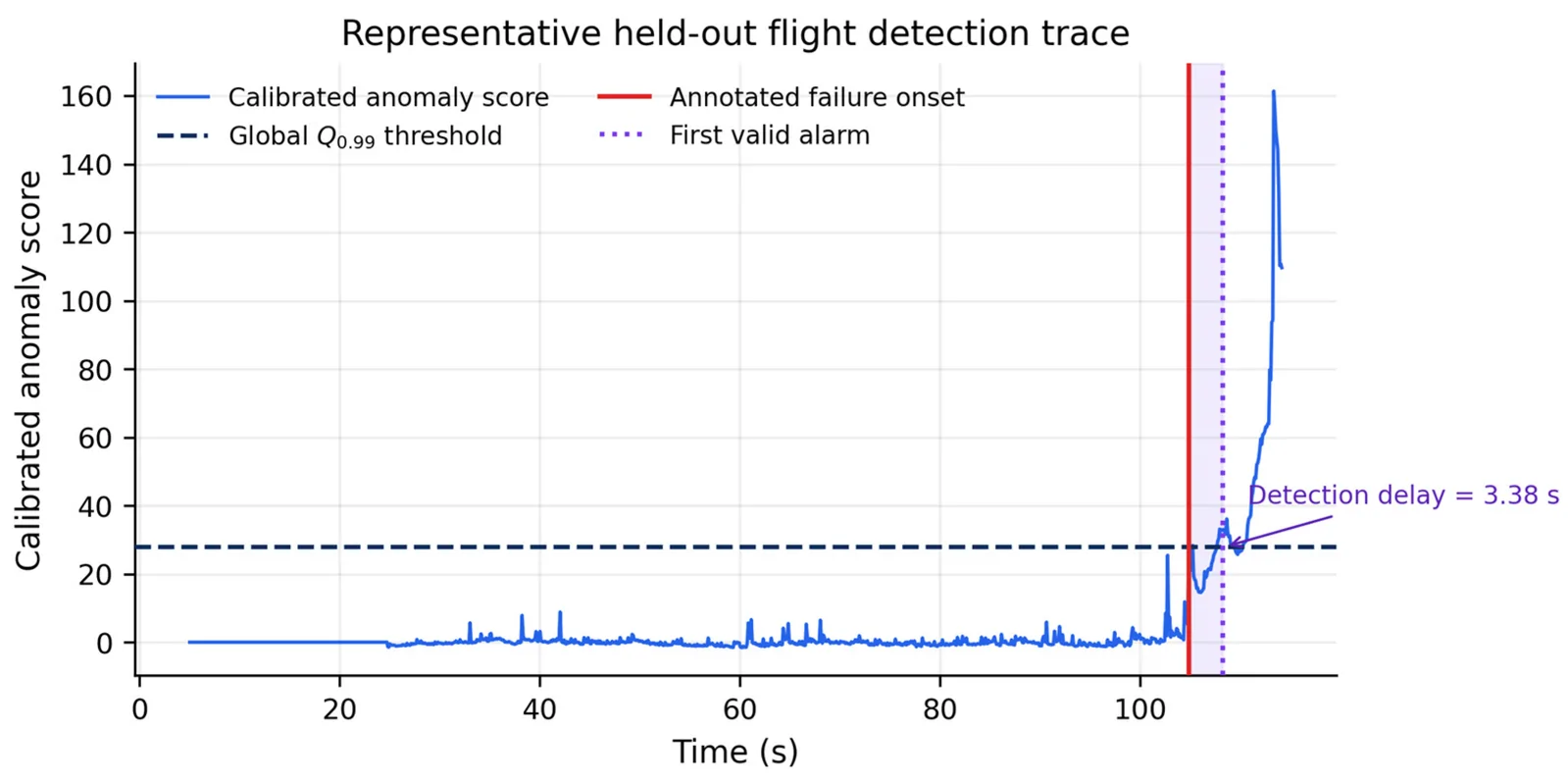
Representative anomaly detection trace for a held-out engine-failure flight under the conservative operating configuration, showing the anomaly score, global $Q_{0.99}$ threshold, failure onset, and first valid alarm.

To complement the aggregate operating-point metrics with a direct visualization of anomaly detection behavior, Figure 5 shows a representative held-out failure-containing flight under the conservative configuration using rolling calibration, a global $Q_{0.99}$ threshold, and 0.5 s temporal persistence. To avoid subjective visual selection, the example was selected deterministically from the successfully detected flight–seed instances as the instance whose detection delay was closest to the pooled median detection delay of 3.38 s. The selected instance corresponds to the engine-failure flight carbonZ_2018-09-11-14-22-07_1_engine_failure evaluated with seed 2.

As shown in Figure 5, the annotated failure onset occurs at 104.85 s, after which the calibrated anomaly score increases and exceeds the global threshold of 27.78. Following application of the 0.5 s persistence rule, the first valid post-onset alarm is generated at 108.23 s, corresponding to a detection delay of 3.38 s. This trace illustrates how the forecasting-error signal is converted into a persistent flight-level alarm on a completely held-out trajectory. The example is provided for interpretability rather than as an additional performance estimate; the quantitative conclusions of the study remain based on the complete flight-disjoint evaluation across all folds and repeated seeds.

### 4.4. Generalization to Unseen Sessions

The main flight-disjoint protocol prevents any individual flight or its overlapping windows from appearing in both training and test partitions; however, different flights recorded within the same date-hour session may occur on opposite sides of a fold boundary. To examine whether shared session characteristics influence the estimated generalization performance, an additional session-disjoint evaluation was performed in which complete session-based clients, rather than individual flights, were held out.

Table 10 compares the two evaluation protocols under the same conservative FedAvg operating configuration. Discrimination performance remained comparable under the more restrictive session-disjoint setting. AUC increased from 0.9123 to 0.9278 and AP from 0.8587 to 0.8679. In contrast, the operational metrics deteriorated substantially. Flight-level detection decreased from 82.78% to 71.11%, while median detection delay increased from 3.38 s to 8.10 s. The false-positive event rate increased moderately from 8.17 to 9.43 events/h. For the session-disjoint evaluation, the 95% flight-bootstrap confidence intervals were [0.8847, 0.9644] for AUC, [0.8151, 0.9141] for AP, [58.33%, 82.78%] for flight-level detection, [5.08, 10.75] s for median detection delay, and [1.87, 20.11] events/h for the false-positive event rate.

These results reveal an important distinction between score-level discrimination and alarm-level generalization. Although the forecasting-error scores retained strong ranking performance when evaluated on entirely unseen sessions, the lower detection probability and longer detection delay indicate that operational alarm behavior is more sensitive to session-level distribution shift than AUC or AP alone would suggest.

## 5. Discussion

The experimental findings provide several insights into the use of federated self-supervised forecasting for UAV anomaly detection. Most importantly, the results indicate that the benefit of the proposed framework should not be assessed solely through conventional discrimination metrics. Under the strictly flight-disjoint evaluation protocol, federated training remained competitive with centralized learning, whereas the operational usefulness of the resulting detector depended strongly on score calibration, threshold scope, and temporal persistence. These observations highlight two distinct aspects of the problem: learning a transferable representation of nominal flight dynamics and converting deviations from those dynamics into actionable alarms.

### 5.1. Federated Learning Under Flight-Disjoint Evaluation

The first major finding is that federated aggregation largely recovered the performance lost when models were trained independently on individual clients. FedAvg achieved an AUC of 0.908 and detected 83.3% of failed flights, compared with an AUC of 0.889 and a detection rate of 65.3% for local-only training. At the same time, its performance remained close to centralized training, which achieved an AUC of 0.904 and a detection rate of 84.4%. The overlapping confidence intervals do not support a claim that federated learning statistically outperforms centralized training; rather, they indicate that federated forecasting can remain competitive while avoiding the central collection of raw flight telemetry.

This result is particularly relevant because evaluation is performed on completely held-out flights rather than randomly partitioned telemetry windows. In UAV telemetry, neighboring windows are strongly temporally correlated and may contain largely overlapping samples. Consequently, random window-level splitting can allow highly similar temporal contexts from the same flight to appear in both training and test sets. The flight-disjoint setting used here imposes a more demanding generalization problem: the model must identify anomalous temporal behavior in flights that did not contribute any windows to model fitting, normalization, calibration, or threshold selection. The competitive performance of FedAvg under this protocol therefore suggests that collaborative learning captures nominal flight dynamics that generalize beyond individual training trajectories rather than relying exclusively on flight-specific temporal patterns.

The session-disjoint experiment further qualifies the interpretation of the main flight-disjoint results. Although the primary protocol eliminates direct flight-level leakage, different flights originating from the same date-hour session can occur in both training and test folds. Such flights may share session-specific characteristics related to operating conditions, sensor offsets, vehicle state, or other unobserved contextual factors. When entire sessions were instead held out, AUC and AP remained comparable to the flight-disjoint results, but flight-level detection decreased by 11.67 percentage points, and median detection delay increased by 4.72 s. The false-positive event rate also increased from 8.17 to 9.43 events/h. This divergence indicates that score-ranking performance is relatively robust to the session-level split, whereas operational alarm behavior is more sensitive to generalization beyond previously observed sessions.

Accordingly, the main flight-disjoint results should not be interpreted as equivalent to generalization across independent operational sessions. The stronger operational performance observed under flight-disjoint evaluation may partly reflect session characteristics shared between training and test flights. At the same time, the preservation of AUC and AP under the stricter session-disjoint protocol suggests that the forecasting model continues to capture anomaly-relevant structure beyond individual recording sessions, although translating this discrimination into timely and reliable alarms becomes more difficult under session-level distribution shift.

The difference between local-only and federated training also illustrates the value of cross-client information sharing when individual clients have limited and heterogeneous telemetry. A local model is restricted to the operating conditions represented in its own failure-free data, whereas federated aggregation exposes the global model indirectly to nominal dynamics observed across multiple clients. This broader representation appears to improve generalization to unseen flights without requiring raw telemetry to leave the local context. Importantly, however, this should not be interpreted as a formal privacy guarantee. The present framework limits raw-data transfer, but model updates may themselves reveal information unless additional mechanisms such as secure aggregation or differential privacy are introduced.

Neither the evaluated FedProx configuration nor personalized fine-tuning provided a clear overall improvement over FedAvg. With μ = 0.01, FedProx produced nearly identical discrimination and operational performance to FedAvg. Because the proximal coefficient was not systematically varied, the present experiment cannot determine whether the absence of improvement reflects the modest session-level heterogeneity of the dataset, the selected μ value, or both. Accordingly, the result should be interpreted only as showing that FedProx did not provide a measurable advantage at μ = 0.01 under the present experimental conditions, rather than as a general conclusion about the effectiveness of proximal regularization. Personalized fine-tuning slightly improved AUC and reduced false-alarm burden, but this improvement was accompanied by increased detection latency and a small reduction in flight-level detection. These results favor the simpler FedAvg configuration for the present dataset, while leaving open the possibility that stronger personalization or heterogeneity-aware optimization may become beneficial in larger fleets with greater differences in airframe type, payload, environment, or mission profile. Accordingly, the present experiments provide stronger evidence that distributed training can approximate centralized learning under the observed session-level statistical heterogeneity than they do for distinguishing among federated optimization strategies under severe client heterogeneity.

These findings can be positioned against recent federated-learning studies in UAV and aviation systems. Previous work has primarily examined federated learning in terms of statistical heterogeneity [4,21], privacy-preserving and communication-efficient aggregation [22,23,24], swarm coordination and federated reinforcement learning [25], unreliable wireless communication [26], distributed object detection [27], non-IID aggregation [28], and heterogeneous representation learning [29]. More recent studies have further integrated federated learning with semantic communication and resource optimization [30], decentralized incentive mechanisms [31], secure split-federated learning [32], privacy-preserving edge infrastructures [33], and mission-level multi-agent coordination [34]. In contrast, the present results examine whether federated learning can preserve the temporal dynamics required for self-supervised UAV telemetry anomaly detection when generalization is measured on completely unseen flights. The finding that FedAvg remained close to centralized training while substantially outperforming local-only learning is consistent with the broader literature showing that collaborative learning can mitigate limitations arising from locally restricted and heterogeneous data [21,28,29]. However, the present study extends this perspective from classification, communication, sensing, and coordination tasks to flight-level anomaly forecasting and explicitly evaluates the resulting detector in terms of detection probability, latency, and false-alarm behavior.

The partial-client-participation experiment provides an additional perspective on the sensitivity of FedAvg to intermittent client availability. Reducing per-round participation from 100% to 75% produced essentially unchanged discrimination and flight-level detection, although the median detection delay increased from 3.38 to 4.17 s and the false-positive event rate increased slightly from 8.17 to 8.49 events/h. With only 50% of eligible clients participating in each round, AUC remained at 0.9117, compared with 0.9123 under full participation, while flight-level detection was 83.33% and the false-positive event rate remained 8.17 events/h. These results indicate that, within the session-partitioned experimental setting considered here, FedAvg was not strongly degraded by random partial participation at the evaluated participation levels. However, this experiment models client availability statistically and should not be interpreted as a simulation of a complete wireless federated UAV network. In particular, it does not capture packet loss, communication latency, bandwidth limitations, asynchronous aggregation, stragglers, or heterogeneous onboard computing resources.

The controlled GRU comparison further indicates that the main observations are not unique to the specific LSTM forecasting backbone. Under identical federated training and alarm-processing conditions, the GRU produced an AUC of 0.9108 compared with 0.9123 for the LSTM, while flight-level detection decreased modestly from 82.78% to 81.11%. The GRU also exhibited a longer median detection delay (4.50 s versus 3.38 s), whereas both architectures produced the same false-positive event rate of 8.17 events/h. Importantly, the 95% flight-bootstrap intervals substantially overlapped for both AUC ([0.860, 0.957] for LSTM and [0.859, 0.955] for GRU) and flight-level detection ([0.706, 0.928] and [0.694, 0.917], respectively). Therefore, the observed point estimates should not be interpreted as evidence that LSTM is statistically superior to GRU. Rather, the similar performance obtained with the alternative recurrent backbone suggests that the principal findings of the federated self-supervised forecasting pipeline are not solely an artifact of the selected LSTM architecture. Nevertheless, this comparison is limited to two recurrent architectures, and broader architectural generalization to TCNs, Transformers, or other modern sequence models remains to be established.

### 5.2. Discrimination Performance Versus Operation Alarm Quality

A second important finding is the clear separation between anomaly score discrimination and operational alarm quality. Across the calibration and feature-ablation experiments, AUC varied only from 0.902 to 0.912, whereas flight-level detection ranged from 80.6% to 96.1% and false-alarm burden ranged from 126.5 to 461.5 events per hour. Thus, relatively small changes in ranking performance were associated with much larger changes in the behavior of the thresholded detector.

The uncalibrated configuration provides the clearest example. Its detection rate of 96.1% is the highest among the ablations, but this apparent improvement is accompanied by 461.5 false-alarm events per hour. In isolation, the detection rate could, therefore, lead to an overly favorable assessment of the detector. Conversely, configurations with slightly lower discrimination or detection may provide substantially more useful operating characteristics once alarm frequency and latency are considered. This finding supports the use of flight-level operational metrics alongside AUC and AP when evaluating anomaly detectors intended for real-time UAV monitoring.

The results also demonstrate that score calibration is not merely a preprocessing detail but a component of the alarm system itself. Rolling calibration increased AUC from 0.908 to 0.912 and detection from 83.3% to 87.8% relative to the warm-up reference while simultaneously reducing the false-alarm burden from 258.5 to 202.1 events per hour. One plausible explanation is that a temporally adaptive reference better compensates for gradual changes in nominal prediction-error magnitude caused by evolving flight conditions. A fixed warm-up reference, by contrast, assumes that the score distribution observed near the beginning of a flight remains representative throughout the mission. The present results suggest that this assumption may be too restrictive for nonstationary UAV telemetry.

At the same time, the feature ablation provides an important check against an artificially favorable result. Including autopilot-derived tracking-error channels did not improve performance; AUC decreased to 0.902 and detection to 80.6%. Because such channels can encode quantities closely related to control failures, excluding them from the primary feature set reduces the risk that the detector simply exploits near-direct failure proxies. The persistence of competitive performance without these channels strengthens the interpretation that the forecasting model is responding to broader multivariate changes in flight dynamics.

The present findings also complement previous telemetry-based UAV anomaly detection studies. Existing approaches have demonstrated the effectiveness of temporal and multivariate modeling through telemetry reconstruction and LSTM-based representations [2], recurrent and attention-based architectures [6], causality-enhanced graph modeling [9], multi-domain feature fusion [10], spatiotemporal representation learning [12], and prediction-based anomaly detection [13]. Related time-series research has likewise shown the usefulness of recurrent and generative modeling for identifying deviations from nominal temporal behavior [18]. The present study follows the same general motivation of learning temporal dependencies from multivariate observations but differs in two important respects. First, the forecasting model is trained collaboratively in a federated setting rather than from a centrally collected telemetry corpus. Second, the evaluation explicitly separates anomaly score discrimination from the behavior of the resulting alarm system. Consequently, the relatively narrow AUC range observed across the present ablations (0.902–0.912), despite much larger variations in failed-flight detection and false-alarm frequency, provides an empirical example of why discrimination-oriented metrics alone may not fully characterize the practical behavior of a UAV anomaly detector. Direct numerical ranking against the above studies is not attempted because their datasets, fault scenarios, learning objectives, partitioning strategies, and reported metrics differ from those used here.

### 5.3. Threshold Scope, Persistence, and Deployment Trade-Offs

The operating-point analysis further shows that alarm policy can have as much practical importance as the underlying forecasting model. Because all points in the final sweep are generated from identical rolling-calibrated FedAvg score streams, the substantial differences in detection, latency, and false-alarm burden arise from threshold and persistence choices rather than model retraining. This distinction is important for deployment because it means that operational behavior can be adjusted to some extent without changing the learned representation.

Replacing client-specific thresholds with a global threshold, for example, reduced false alarms from 202.1 to 66.0 events per hour without persistence, while detection decreased only from 87.8% to 87.2% and median delay increased from 0.44 to 0.72 s. Adding 0.3 s persistence to the personal-threshold configuration reduced the alarm burden further to 27.0 events per hour while retaining 86.1% detection, although median delay increased to 1.75 s. More aggressive persistence continued to suppress alarms, reaching 7.5 events per hour for the global threshold with 1.0 s persistence, but detection fell to 81.7%, and median delay increased to 3.92 s.

These results illustrate why there is no universally optimal operating point. A low-latency monitoring application may tolerate a larger number of alarms in exchange for rapid detection, whereas an unattended system may require much stronger false-alarm suppression even if this increases detection latency. The appropriate configuration, therefore, depends on mission-specific costs associated with missed detections, delayed responses, and unnecessary interventions. Rather than optimizing one metric independently, UAV anomaly detection systems should be configured by jointly considering these competing operational objectives.

The operating-point analysis reinforces this limitation. Reducing the false-alarm burden from 66.0 to 8.17 events/h increased event precision from 0.428 to 0.851 while retaining 82.8% flight-level detection but increased the median detection delay from 0.72 to 3.38 s. A more conservative configuration reached 4.40 events/h and an event precision of 0.903, but detection decreased to 72.2%, and the median delay increased to 6.05 s. Moreover, none of the 56 evaluated configurations achieved the analytical target of ≤1 false-positive event/h. These results demonstrate that false-alarm suppression cannot be considered independently of missed detections and detection latency.

Nevertheless, none of the evaluated configurations should be interpreted as deployment-ready for an unattended safety-critical warning system. Even the more conservative operating points produce a non-negligible alarm burden, and stronger persistence reduces this burden partly by delaying detection and missing additional failed flights. The current results therefore establish the feasibility of federated flight-level anomaly detection and expose the operational trade-offs that must be addressed, rather than demonstrating that the resulting detector is ready for autonomous safety intervention. This interpretation is consistent with the operational limitations observed in the final operating-point analysis.

The practical implications of the observed false-alarm rates can also be expressed in terms of alarm frequency over time. A burden of 66.0 false-positive events/h corresponds to approximately one false alarm every 55 s of nominal exposure, which would require nearly continuous operator attention and is therefore unsuitable for unattended monitoring. The more conservative operating point of 8.17 events/h corresponds to approximately one false alarm every 7.3 min, while 4.40 events/h corresponds to approximately one every 13.6 min. Although these reductions substantially improve the practical alarm burden, they still represent recurring false alerts during nominal operation and may remain excessive for long-duration or minimally supervised UAV missions. Importantly, the analytical targets of ≤10, ≤5, and ≤1 false-positive event/h used in this study are not claimed as established UAV certification or industry requirements; rather, they provide progressively stricter reference levels for quantifying the detection–latency–false-alarm trade-off. Application-specific acceptable alarm rates will depend on factors such as mission duration, level of autonomy, operator workload, fleet size, and the consequence of missed detections.

From a broader evaluation perspective, this operating-point analysis also distinguishes the present study from much of the related UAV anomaly detection literature. Previous telemetry-based studies have primarily characterized detector performance using sample-, window-, or fault-recognition-oriented measures [2,6,9,10,11,12,13,14,15,16,17,18,19], while recent flight-level work has emphasized the importance of preventing highly correlated windows from the same flight from being shared across training and test partitions [7]. The present evaluation combines this flight-disjoint principle with alarm-level measures that quantify not only whether a failed flight is detected but also when the first valid alarm occurs, how frequently false-positive events are generated during nominal exposure, and what proportion of generated alarm events correspond to detected failures. This distinction is consequential in the present experiments: configurations with similar discrimination performance produced markedly different detection delays and false-alarm burdens. The comparison therefore suggests that methodological progress in UAV anomaly detection should be assessed not only through improvements in conventional predictive metrics but also through evaluation protocols that reflect complete-flight generalization and the operational consequences of thresholded alarm generation.

### 5.4. Limitations and Implications for Future Systems

Several limitations should be considered when interpreting these findings. First, the evaluation is based on 47 flights from the ALFA corpus, including 36 failure-containing and 11 nominal flights. Although flight-disjoint cross-validation reduces leakage and provides a more realistic estimate of generalization than random window splitting, the number of independent flight trajectories remains limited. Accordingly, the present findings should be interpreted as evidence obtained from a controlled benchmark rather than as population-level estimates of performance across UAV operations, and validation on larger datasets containing more independent missions, UAV platforms, operating environments, and failure conditions is required to determine the broader generalizability of the observed results.

The additional session-disjoint evaluation further shows that flight-disjoint separation alone does not ensure independence at the session level. While score-level discrimination remained stable when complete sessions were held out, the reduction in flight-level detection and increase in detection latency indicate sensitivity to session-level distribution shifts. Because the ALFA corpus contains only 13 session proxies, the session-disjoint estimates also carry substantial uncertainty, as reflected in the relatively wide bootstrap intervals. Evaluation on larger collections containing independently recorded sessions, different UAV platforms, and broader operating conditions is therefore required to characterize cross-session generalization more reliably.

Second, the 13 federated clients represent temporally coherent recording sessions constructed from a single corpus rather than physically independent UAVs or operators. This client definition provides a controlled mechanism for evaluating parameter aggregation across statistically heterogeneous local partitions, but the resulting heterogeneity arises primarily from differences in session composition and local data volume. It therefore does not reproduce the broader sources of heterogeneity that would be expected among independent UAVs, such as differences in airframe dynamics, payloads, sensor characteristics, onboard hardware, mission profiles, environments, and operator-specific data distributions. Consequently, the observed similarity between federated and centralized performance should be interpreted as evidence that distributed parameter aggregation is effective under the session-level statistical heterogeneity represented in ALFA, rather than as evidence that equivalent performance would necessarily be obtained across a heterogeneous real-world UAV fleet. The session-disjoint results reinforce this distinction by showing that operational detection performance degrades when generalization is extended from unseen flights to entirely unseen sessions. Although intermittent client availability is examined through the controlled partial-participation experiment, communication loss, asynchronous updates, bandwidth constraints, stragglers, and device-specific computational limitations are also not explicitly modeled. These factors may further alter convergence, communication cost, and anomaly detection performance in an operational federated deployment.

Third, the present study evaluates anomaly detection rather than failure isolation. The forecasting error indicates that observed telemetry has deviated from learned nominal dynamics, but the detector does not identify whether the underlying cause is an engine, aileron, rudder, elevator, sensor, or environmental anomaly. This distinction is important for autonomous response because different failure modes may require different recovery actions. Extending the framework from detection to fault isolation, therefore, represents a natural next step.

Finally, keeping raw telemetry local reduces the need to centralize potentially sensitive flight logs but does not by itself provide formal privacy protection. Model updates can remain vulnerable to inference or reconstruction attacks. Future federated UAV monitoring systems should therefore investigate secure aggregation, differential privacy, and communication-efficient update mechanisms together with their effects on anomaly detection performance. Such extensions should be evaluated under the same flight-disjoint and operationally oriented methodology used here so that gains in privacy or communication efficiency are not obtained at the expense of unacceptable detection latency or alarm burden.

## 6. Conclusions and Future Work

This study investigated federated self-supervised forecasting for UAV flight anomaly detection under a strictly flight-disjoint evaluation protocol. Rather than randomly partitioning highly overlapping telemetry windows, complete flights were held out from model training, normalization, calibration, and threshold estimation, providing a more stringent assessment of generalization to unseen flight trajectories. Local-only training, FedAvg, FedProx, personalized FedAvg, and centralized learning were compared, while the effects of anomaly score calibration, threshold scope, potentially leaky autopilot-derived channels, and temporal alarm persistence were examined separately.

Within the session-partitioned experimental setting considered in this study, the results show that federated forecasting can remain competitive with centralized learning without requiring raw flight telemetry to be transferred to a common training repository. FedAvg achieved an AUC of 0.908 and detected 83.3% of failed flights, compared with an AUC of 0.904 and an 84.4% detection rate for centralized training. In contrast, local-only training achieved lower discrimination and detected only 65.3% of failed flights. Neither FedProx at the evaluated μ = 0.01 nor the tested personalized fine-tuning configuration provided a clear overall advantage over FedAvg under the present experimental conditions, supporting the use of the simpler FedAvg configuration as the backbone for the subsequent analyses.

More importantly, the experiments demonstrate that discrimination performance alone is insufficient for assessing an operational anomaly detector. Calibration and alarm-policy choices produced substantial changes in detection probability, latency, and false-alarm burden despite relatively small differences in AUC. Rolling calibration provided the most suitable score-processing configuration among those evaluated, while global thresholding and temporal persistence substantially reduced false-alarm frequency at the cost of varying degrees of detection loss and additional latency. For example, under rolling calibration, replacing a personal threshold with a global threshold without persistence reduced the alarm burden from 202.1 to 66.0 events per hour while retaining 87.2% flight-level detection with a median delay of 0.72 s. The operating-point analysis showed that the false-alarm burden could be reduced to 8.17 events/h while retaining 82.8% failed-flight detection and an event precision of 0.851, although the median detection delay increased to 3.38 s. A more conservative configuration reached 4.40 events/h and an event precision of 0.903, but detection decreased to 72.2% and median delay increased to 6.05 s. No evaluated configuration achieved the analytical target of ≤1 false-positive event/h. These results further demonstrate that false-alarm suppression is accompanied by increasing detection latency and missed failed flights.

These findings lead to two main conclusions. First, federated self-supervised forecasting is a viable approach for learning transferable nominal UAV telemetry dynamics without centralizing raw flight data. Second, model-level discrimination metrics such as AUC and AP should not be treated as sufficient evidence of operational readiness. Evaluation should additionally consider complete unseen flights and jointly report detection probability, detection latency, and false-alarm burden. In this respect, the present results demonstrate the feasibility of the proposed approach rather than a deployment-ready safety-warning system.

Future work will focus on two immediate extensions of the present study. First, the framework should be evaluated on larger and more diverse collections of independent flights to determine whether the observed flight-disjoint generalization and detection–false-alarm trade-offs remain consistent across different UAV platforms, mission profiles, and failure conditions. Second, the current anomaly detection framework should be extended toward failure isolation, enabling detected anomalies to be associated with specific failure types such as engine, aileron, rudder, or elevator faults. This extension would move the framework beyond identifying abnormal flight behavior toward providing failure-specific information that could support subsequent diagnosis and recovery decisions. These two directions directly address the principal limitations of the current evaluation and provide a focused path toward more reliable flight-level UAV health monitoring.

## Figures and Tables

**Table 1:** Summary of related research directions and limitations with respect to the present study.

Research Direction	Representative References	Main Focus	Limitation Relative to the Present Study
Telemetry-driven UAV anomaly and fault detection	[2,6,9,10,11,12,13,14,15,16,17,18,19]	Temporal modeling, reconstruction, prediction, multimodal analysis, and fault recognition	Evaluation is predominantly based on samples, windows, specific fault scenarios, or fault-recognition performance; complete unseen-flight generalization and operational alarm behavior receive less attention.
UAV operational reliability and adaptive operation	[3,20,43,44,45,46,47]	Mission reliability, health management, uncertainty, digital twins, communication, and deployment constraints	Operational reliability is considered, but not jointly with federated self-supervised telemetry forecasting under flight-disjoint evaluation.
Federated learning for UAV and aviation systems	[4,21,22,23,24,25,26,27,28,29,30,31,32,33,34]	Non-IID learning, aggregation, communication efficiency, distributed optimization, and coordination	The principal tasks are generally classification, communication/resource optimization, object recognition, or policy learning rather than self-supervised flight anomaly forecasting.
Federated UAV security and privacy	[5,35,36,37,38,39,40,41,42]	Privacy, poisoning defense, secure aggregation, intrusion detection, and adversarial robustness	Security and privacy are emphasized, while flight-level anomaly generalization, detection latency, and false-alarm behavior are not jointly evaluated.
Present study	This work	Federated self-supervised telemetry forecasting with strict flight-disjoint evaluation	Jointly evaluates unseen-flight discrimination, failed-flight detection, detection delay, event precision, and false-alarm burden.

**Table 2:** Dataset and evaluation summary.

Property	Value
Flights	47
Failed/nominal flights	36/11
Samples/sampling rate	49,165/10 Hz
Total duration	81.9 min
Primary input channels	52
Federated clients	13 date-hour session proxies
Cross-validation	3-fold, flight-disjoint
Repeated seeds	5

**Table 3:** Model architecture and training hyperparameters used in the main experiments.

Parameter	Value
Sampling frequency	10 Hz
Window length L	50 samples (5 s)
Window stride	1 sample
Input dimension d	52
LSTM layers	1, unidirectional
Hidden dimension	64
LSTM dropout	0.0
Output layer	Linear, 64 → 52
Output activation	None
Forecasting loss	Mean squared error
Optimizer	Adam
Learning rate	$10^{-3}$
Batch size	256
Local epochs per round	2
Federated rounds R	20
FedProx coefficient μ	0.01
Fine-tuning epochs	5
Fine-tuning learning rate	$3\times 10^{-4}$

**Table 4:** Main comparison across five seeds with flight-level bootstrap confidence intervals (36 failure-containing flights).

Method	AUC (95% CI)	AP (95% CI)	Detection	Median Delay	False Alarms/h
Local	0.889 [0.825, 0.945]	0.791 [0.695, 0.877]	0.653	2.67 s	43.1
FedAvg	0.908 [0.856, 0.954]	0.816 [0.732, 0.891]	0.833	0.47 s	258.5
FedProx	0.907 [0.854, 0.953]	0.816 [0.732, 0.892]	0.833	0.47 s	257.1
FedAvg + fine-tuning	0.910 [0.859, 0.954]	0.818 [0.735, 0.892]	0.828	1.45 s	193.2
Centralized	0.904 [0.862, 0.940]	0.762 [0.687, 0.828]	0.844	1.75 s	236.7

**Table 5:** Effect of partial client participation on FedAvg under flight-disjoint evaluation.

Participation	AUC	AP	Detection (%)	Median Delay (s)	FP Events/h
100%	0.9123	0.8587	82.78	3.38	8.17
75%	0.9123	0.8565	82.78	4.17	8.49
50%	0.9117	0.8565	83.33	3.48	8.17

**Table 6:** Controlled comparison of LSTM and GRU forecasting backbones under the same federated and alarm configuration.

Model	AUC	AP	Detection (%)	Median Delay (s)	FP Events/h
LSTM	0.9123	0.8587	82.78	3.38	8.17
GRU	0.9108	0.8473	81.11	4.50	8.17

**Table 7:** Effect of score calibration, threshold scope, and autopilot-derived tracking-error channels on FedAvg performance.

FedAvg Condition	AUC	Detection	Median Delay	False Alarms/h
Warm-up, personal threshold, no leaky channels	0.908	0.833	0.47 s	258.5
Rolling, personal threshold	0.912	0.878	0.44 s	202.1
No calibration	0.909	0.961	0.32 s	461.5
Warm-up, global threshold	0.909	0.817	1.75 s	126.5
Warm-up, leaky channels	0.902	0.806	1.51 s	221.4

**Table 8:** Representative operating points under rolling-calibrated FedAvg.

Threshold Scope	Persistence	Detection Rate	Median Delay	False Alarms/h
Personal	0.0 s	87.8%	0.44 s	202.1
Global	0.0 s	87.2%	0.72 s	66.0
Personal	0.3 s	86.1%	1.75 s	27.0
Personal	0.5 s	85.0%	2.27 s	18.2
Global	1.0 s	81.7%	3.92 s	7.5

**Table 9:** Conservative operating-point analysis for the rolling-calibrated FedAvg detector.

Analytical Target/Reference	Threshold Scope	Quantile	Persistence	Detection (%)	Median Delay (s)	TP Events	FP Events	FP Events/h	Event Precision
Low-latency reference	Global	0.990	0.0 s	87.2	0.72	157	210	66.00	0.428
Selected at ≤10 FP/h	Global	0.990	0.5 s	82.8	3.38	149	26	8.17	0.851
Selected at ≤5 FP/h	Global	0.995	2.0 s	72.2	6.05	130	14	4.40	0.903
≤1 FP/h	-	-	-	-	-	-	-	Not achieved	-

**Table 10:** Comparison of flight-disjoint and session-disjoint evaluation under the same conservative FedAvg operating configuration.

Evaluation Protocol	AUC	AP	Detection (%)	Median Delay (s)	FP Events/h
Flight-disjoint	0.9123	0.8587	82.78	3.38	8.17
Session-disjoint	0.9278	0.8679	71.11	8.10	9.43

## Data Availability

The data used in this study are from the publicly available AirLab Failure and Anomaly (ALFA) dataset developed in [8]. The dataset, documentation, and download information are available from the official ALFA dataset website: https://theairlab.org/alfa-dataset, accessed on 27 September 2026. No new flight dataset was collected as part of this study. The source code used to implement the leakage-resistant flight- and session-disjoint evaluation protocols, preprocessing pipeline, federated training procedures, anomaly score calibration, threshold selection, alarm persistence and event-merging logic, and evaluation metrics is publicly available on Zenodo at https://doi.org/10.5281/zenodo.22995000.

## Abbreviations

The following abbreviations are used in this manuscript:

ALFA	AirLab Failure and Anomaly
AP	Average Precision
AUC	Area Under the Curve
CI	Confidence Interval
CNN	Convolutional Neural Network
FedAvg	Federated Averaging
FedProx	Federated Proximal
GRU	Gated Recurrent Unit
LSTM	Long Short-Term Memory
MAVROS	MAVLink extendable communication node for ROS
ROC	Receiver Operating Characteristic
UAV	Unmanned Aerial Vehicle
